# Impacts of innovation in dental care delivery and payment in Medicaid managed care for children and adolescents

**DOI:** 10.1186/s12913-021-06549-3

**Published:** 2021-06-08

**Authors:** Douglas A. Conrad, Peter Milgrom, Yuxian Du, Joana Cunha-Cruz, Sharity Ludwig, R. Mike Shirtcliff

**Affiliations:** 1 Department of Health Services, School of Public Health, University of Washington, Box 357660, Seattle, WA USA; 2 Department of Oral Health Sciences, School of Dentistry, University of Washington, Box 357475, Seattle, WA 98195-7475 USA; 3 Fred Hutchison Cancer Research Center, Seattle, WA USA; 4 Advantage Dental Services LLC, Redmond, OR USA; 5 RMS Dental Director, Inc, 6860 Thunderbird Ct., Redmond, OR USA

**Keywords:** Innovation, Hub and spoke model, Medicaid, Value-based payment

## Abstract

**Background:**

We evaluated a 14-county quality improvement program of care delivery and payment of a dental care organization for child and adolescent managed care Medicaid beneficiaries after 2 years of implementation.

**Methods:**

Counties were randomly assigned to either the intervention (PREDICT) or control group. Using Medicaid administrative data, difference-in-difference regression models were used to estimate PREDICT intervention effects (formally, “average marginal effects”) on dental care utilization and costs to Medicaid, controlling for patient and county characteristics.

**Results:**

Average marginal effects of PREDICT on expected use and expected cost of services per patient (child or adolescent) per quarter were small and insignificant for most service categories. There were statistically significant effects of PREDICT (*p* < .05), though still small, for certain types of service:

1. Expected number of diagnostic services per patient-quarter increased by .009 units;
2. Expected number of sealants per patient-quarter increased by .003 units, and expected cost by $0.06;
3. Total expected cost per patient-quarter for all services increased by $0.64.

These consistent positive effects of PREDICT on diagnostic and certain preventive services (i.e., sealants) were not accompanied by increases in more costly service types (i.e., restorations) or extractions.

**Conclusion:**

The major hypothesis that primary dental care (selected preventive services and diagnostic services in general) would increase significantly over time in PREDICT counties relative to controls was supported. There were small but statistically significant, increases in differential use of diagnostic services and sealants. Total cost per beneficiary rose modestly, but restorative and dental costs did not. The findings suggest favorable developments within PREDICT counties in enhanced preventive and diagnostic procedures, while holding the line on expensive restorative and extraction procedures.

**Supplementary Information:**

The online version contains supplementary material available at 10.1186/s12913-021-06549-3.

## Background

### Overview

Access to dental care has improved for children from low-income families in the United States in recent decades [1]. These changes have occurred because of Medicaid expansion, growth of Federally Qualified Health Centers, and development of private and not-for-profit dental care organizations [2]. Access nevertheless remains uneven with rural areas having lower rates of care than areas with greater population density [3].

Delivery system changes, in which many Medicaid recipients are now served by dental care organizations (DCOs), have created an opportunity to test hub and spoke models [4]. In such models, programs in schools and community facilities provide basic care delivered by expanded practice dental hygienists (EPDHs) or therapists and refer complex care to hubs staffed by general dentists and specialists.

In parallel with delivery changes, protocols have been developed to lower disease levels. Examples include using caries risk status to determine the amount and intensity of preventive and restorative care including fissure sealants and application of topical fluorides and silver diamine fluoride to prevent or arrest developing dental caries lesions [5, 6]. Hub and spoke models potentially could implement these evolving care protocols efficiently [4, 5].

In this paper, we examine the quality improvement initiative of one DCO that developed a hub and spoke model in rural areas where it had substantial market penetration. This study analyzes intervention effects on utilization and cost of dental services for children and adolescents. This intervention, The Population-centered Risk- and Evidence-based Dental Interprofessional Care Team (PREDICT), was part of the Robert Wood Johnson Foundation Finding Answers: Solving Disparities through Payment and Delivery System Reform program [7].

## Methods

### Setting

The DCO, Advantage Dental Services (ADS), was a dentist-owned, limited liability corporation. It delivered services to almost 300,000 Medicaid enrollees, primarily in rural Oregon state, U.S.A. through more than 50 staff model clinics and over 1100 contracted primary care and specialist dentists. The quality improvement (QI) project and evaluation included children and adolescents (≦18) assigned by Oregon Health Authority (OHA) to the DCO.

Oregon state is on the vanguard of oral health care transformation in the U.S.A. Since 2012 the state’s Medicaid purchasing authority has paid each coordinated care organization (CCO) a global budget capitation per member per month (PMPM). In turn, each CCO makes PMPM payments to providers of oral health services (POHs) [8]. ADS is one of those contracted POHs.

The PREDICT innovation exemplifies value-based care delivery and payment (VBP) models that are being designed to improve access, enhance quality, and lower cost in oral health care [9, 10].

### The hub and spoke model

PREDICT was organized to improve access and reduce dental caries for clients in 14 counties. The study population was Medicaid-enrolled children and adolescents ages 0–18. The QI was financed by research and development funds from ADS’ global budget.

The delivery system was implemented in January 2016 in six randomly chosen test counties. In PREDICT, salaried EPDHs provided screening, risk assessment and preventive care, including non-restorative treatment of carious lesions^1^ in community settings [11]. Care provided by EPDHs was determined by clinical algorithms [6]. Primary care dentists (PCDs) contracted with ADS had access to the electronic dental record (EDR). Regional Manager Community Liaisons were responsible for agreements permitting EPDHs to service community settings. Case managers, who were centralized, served as patient navigators to arrange referrals for services at hub practices.

In eight control counties ADS did not change its existing delivery system or payment incentives. Care was primarily delivered by ADS-contracted practices (PCDs) and staff dentists in ADS-owned clinics but some pre-existing school-based services for screening, application of fluoride varnish, sealants, and dental health education were maintained.

### Compensation model

ADS had a bonus system for all 14 counties focused on primary care owner dentists to encourage increasing access. Also, owner dentists shared yearly company profits. PCDs were paid a capitation fee per member per month and dentists employed by company owned practices were salaried.

For employees in PREDICT counties, new QI metrics focused on improving access for children and gave bonuses based on quarterly performance. Dentists and employees in PREDICT counties were subject to financial incentives based on Medicaid members’ access to dental services, preventive treatment (topical fluoride) for children and adolescents, and care within 60 days to high-risk children with urgent need [9]**.**

### Data sources

ADS enrollment files were sent to OHA by secure transfer protocol, and OHA added eligibility and claims data. ADS stripped OHA identifiers of each member, replaced them with a masked identifier, and sent the de-identified dataset to the study team. County-level variables for dentist/population ratio [12], percent medically uninsured [13], and population density [14] were derived from state reports.

### Dependent variables measuring utilization and cost

Utilization and cost were measured per person per quarter to capture seasonal variation and to increase the granularity of analysis of utilization and cost over time. The four-year observation period was 2014–2017; 2014–2015 was the baseline (pre-intervention) period, and 2016–2017 was the intervention period.

The following services are the study’s dependent measures: any service (CDT D0000-D9999), preventive (D1000–1999), fluoride varnish (D1206), preventive silver diamine fluoride without fluoride varnish (D1208), sealant (D1351), caries arrest (D1354); diagnostic (D0001–0999); restorative (D2000–2999); and extractions (D7111, D7140).

Specific service utilization was measured as a count variable per quarter. Each distinct, billable service or procedure received a count of 1 in the utilization measure. Costs per quarter were expressed as shadow costs, i.e., what a given service would have cost Medicaid if the allowed fee were the actual transaction price. Shadow costs were imputed from the OHA-allowed fee schedule in 2018 dollars. Services not in the schedule were assigned the average allowed fee for their service category (e.g., preventive, restorative). Since shadow costs were fixed over time, changes in shadow cost over time reflect changes in volume of use of each service and mix of services within category (total units of service and in their unit costliness), not general dental price inflation.

### Independent variables

To adjust for potential differences between PREDICT and control counties in personal and area-level determinants of utilization and cost, the following covariates were included in all statistical models: age (0–5, 6–12, and 13–18), gender, self-reported race/ethnicity (White, Hispanic non-White, and Other), days of coverage during the quarter (0–29, 30–59, 60–90), and county characteristics (dentist/population ratio, % medically uninsured, population density per square mile).

### Analytic plan

The evaluation plan was to estimate the effect of PREDICT on utilization and cost of services. The principal hypothesis was that PREDICT might increase primary dental care costs, but could reduce total care costs. The underlying thesis was that by increasing access to services in general (expressed as increased use of any dental services and, in particular, preventive and diagnostic services), PREDICT could decrease utilization of expensive restorative and extraction services. Accordingly, the model estimates PREDICT effects on utilization and costs of specific services.

The basic statistical model is:

Equation (1): Utilization and Cost = f (gender, age, race/ethnicity, length of dental benefit coverage, county characteristics, indicator for residing in PREDICT (intervention) county, a time indicator for pre- vs. post intervention period, interaction term for residing in PREDICT county*post-intervention time period, random error). The interaction term coefficient estimates the effect of the PREDICT intervention.

This analysis employs a difference-in-differences (DID) approach [15–17]. Stata Release 15 was used for all analyses, and the command “vce(cluster)” was used to calculate robust standard errors adjusted for within-county correlation and heteroskedasticity [18]. The intervention effect is measured as change in utilization and cost per patient per quarter from pre-to-post intervention in PREDICT versus control counties. Because of the small number of counties, and spillover effects between adjacent PREDICT and control counties were possible, DID in individual enrollee-level analyses, including available county-level characteristics, was used to eliminate omitted variable bias due to unobserved differences between PREDICT and control counties in enrollee characteristics that do not change over time.

**Fig. 1:**
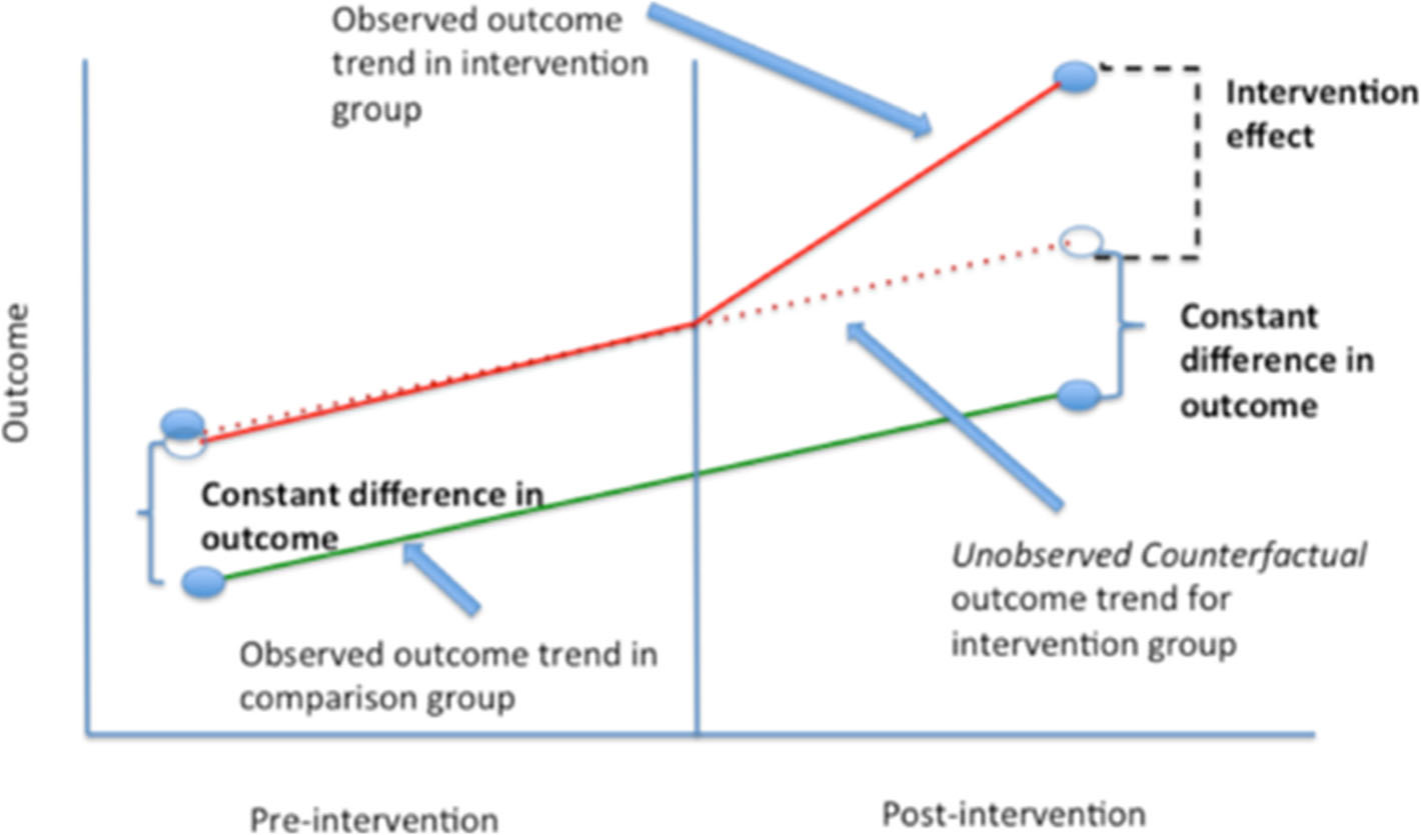
Estimating Intervention Effects in Difference-in-Differences. Source: Columbia Public Health, Difference-in-difference estimation: the parallel trends assumption. URL: https://www.publichealth.columbia.edu/research/population-health-methods/difference-difference-estimation

Figure 1 illustrates the logic of the DID approach in estimating intervention effects. Intervention effects are estimated by subtracting change over time in the outcome of interest in the control group from its change over time in the intervention group. The control group is chosen to reflect what would have happened in the intervention group if it were not subject to the intervention (the “unobserved counterfactual” in Fig. 1).

#### Parallel trend testing

To produce unbiased estimates of PREDICT effects, DID assumes that baseline trends in utilization and cost of the PREDICT and control groups are parallel. This test is required to rule out confounding by omitted factors that vary over time, differ between the intervention and control groups. and which affect the dependent variable [17]. The detailed results of those tests are in Additional file 1 (Figures FA1, FA2, and FA3). We perform a more clearcut test for baseline parallel trends, which uses a linear term for quarter time (valued from 1 to 8), includes season dummies and all other covariates in Eq. (1). The test for parallel baseline linear trends (the null hypothesis) is based on the interaction between the intervention group dummy and the linear time variable. If this interaction is statistically significant (*p* < .05), the null hypothesis of parallel trends is rejected. In Table 5 columns (1), and (5) the results of this test are displayed for the expected nine utilization and expected cost variables, respectively.

#### Regression model functional forms

Estimates of PREDICT effects on outcomes were based on zero-inflated negative binomial regression (ZINB) for count variables of dental service utilization, which account for large numbers of zero utilization [19], and two-part regression models (TPM) for dental services cost variables [20]. ZINB and TPM produce more robust and efficient estimates of expected utilization counts and expected costs, respectively, by combining estimates of probability of any utilization or cost with estimates of level of utilization or cost, given any utilization or cost.

ZINB analyses deployed logistic regression for the probability of utilization portion and a negative binominal model for the count portion (level of utilization, given positive utilization). TPM regressions also estimated logistic models for probability of any cost and generalized linear model (GLM) for level of cost, given positive cost. GLM was based on a log link and gamma distribution. Specification tests revealed ZINB models of expected utilization to be superior to the alternative of zero-inflated Poisson. Deb and Norton, in their review paper on health care utilization and expenditures, have summarized the superiority of these models over ordinary least squares and other techniques [21].

#### Regression model specifications

Regression analyses used four specifications to identify PREDICT effects. To estimate the average PREDICT effect over the entire intervention period, the first specification (the” All” model) of Eq. (1) uses a single PREDICT*Post-Intervention Period interaction term.. The effect of PREDICT is estimated as the average marginal effect**,** following the model specified in Eq. (1), computed as the average difference between individuals in the PREDICT and control group, respectively, of individual estimates over the entire study sample. This model matches the DID model specification used by Card and Krueger in their classic study of minimum wage effects [22]. Those results are presented in columns (2) and (6) for each dental service type in Table 5 in the Results section.

The second regression specification (“R_Full”) adds a vector of “seasonality” dummy variables to the Eq. (1) model, in order to adjust for seasonal variations in dental utilization over the seasons of the year. By including dummies for seasonality, the estimate of PREDICT’s average effect is not distorted by independent quarterly (seasonal) variations over the calendar year. Those “R_Full” model estimates are presented in columns (3) and (7) of Table 5.

The third regression specification (“R_Reduce”) is included as a general check on the sensitivity of our average marginal effect estimates to the inclusion of covariates. This model excludes all covariates, except the seasonality dummies. If randomization of counties to the PREDICT intervention had produced a perfectly balanced set of individual and county characteristics between the PREDICT and control counties (and thus eliminated any confounding), one would expect the PREDICT average marginal effect estimates to be identical for the R_Full and R_Reduce specifications. The R_Reduce model estimates are presented in Columns (4) and (8) of Table 5.

**Fig. 2:**
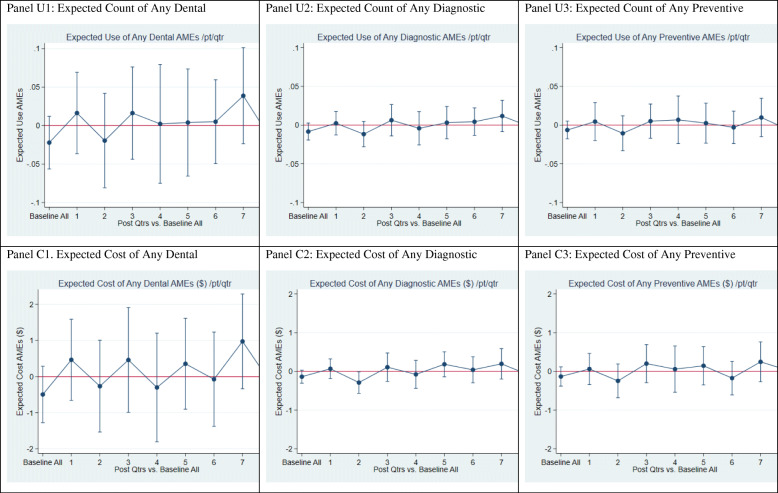
Average Marginal Effect on Any Dental, Diagnostic, and Preventive Services (Post-vs. baseline)

**Fig. 3:**
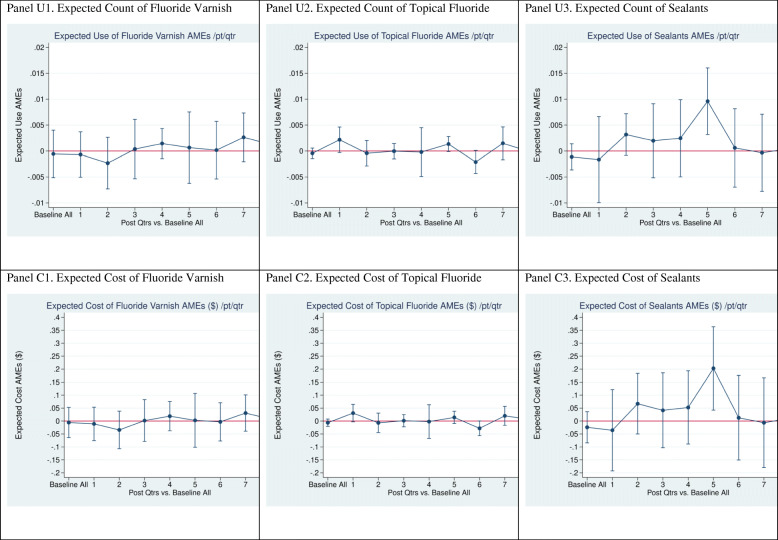
Average Marginal Effect on Fluoride Varnish, Topical Fluoride, and Sealants (Post- vs. baseline)

**Fig. 4:**
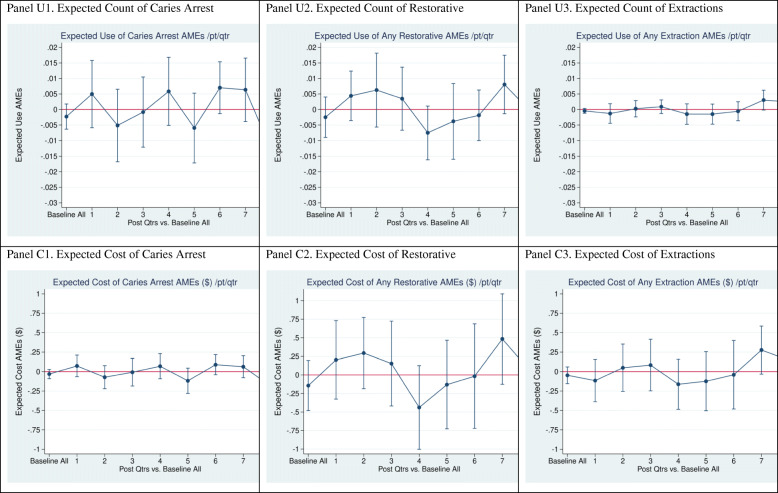
Average Marginal Effects on Caries Arrest, Restorative, and Extractions (Post-intervention vs. baseline)

In a fourth regression specification, PREDICT effects in each post-intervention quarter are estimated, using eight unique interaction terms of PREDICT*Intervention Quarter. Those interactions isolate PREDICT differential effects over time relative to the baseline period (DID). This specification allows one to disaggregate the impact of PREDICT over time. The estimated average marginal effects of PREDICT on dental services utilization and cost by quarter from this specification are displayed in Figs. 2, 3, and 4 in the Results section.

## Results

Study results are presented in two parts. The first displays unadjusted descriptive data for children and adolescents (age ≦18), distinguishing between PREDICT and control counties. The second presents findings of regression analyses of impacts of PREDICT on utilization and costs.

### Descriptive results

#### Beneficiary demographics and county-level characteristics

Table 1 shows that distributions of age, gender, length of Medicaid dental coverage, and county-level percent medically uninsured are quite similar between PREDICT and control counties at baseline and remain so during the intervention. While statistically significant, differences in length of coverage and percent uninsured are quite small and not likely to be practically meaningful. In contrast, race/ethnicity, county-level dentists per 100,000 population, and population density do differ between PREDICT and control counties. The percentage of white youth is roughly 10 percentage points greater in PREDICT counties. The dentist-to-population ratio is approximately 15 percentage points higher in the PREDICT counties, and population density in PREDICT counties is more than double that of control counties (18 percentage points higher). Clearly, PREDICT counties are less diverse in their race/ethnicity, denser in population, and greater in dentist supply relative to population. These differences, potentially important for dental outcomes, are included as covariates in the regression models and are relatively stable over time. Thus, validity of our DID estimates of PREDICT effects is not likely compromised by these differences.

**Table 1:** Individual sociodemographic characteristics and county-level characteristics

Year	Baseline Period (2014–2015)			Intervention Period (2016–2017)			Notes
*Group*	Control	Intervention	*p*-value	Control	Intervention	*p*-value	
*Sample Size (N)*	61,743	124,013		52,497	119,170		
**Age in years**							
0 to 5	37.82%	37.27%	0.0511	35.62%	35.05%	0.0504	[1]
6 to 12	36.00%	36.18%		37.49%	37.64%		
13 to 18	26.18%	26.55%		26.89%	27.31%		
**Gender**							
Female	49.16%	48.84%	0.2014	49.00%	48.79%	0.4317	[1]
Male	50.84%	51.16%		51.00%	51.21%		
**Race/Ethnicity**							
White	58.84%	68.08%	**<.0001**	55.23%	66.27%	**<.0001**	[1]
Hispanic Non-White	30.14%	21.60%		31.02%	20.93%		
Other	11.03%	10.32%		13.75%	12.80%		
**Coverage Days category**							[2]
< 30 days	0.80%	0.60%	**<.0001**	0.95%	0.71%	**<.0001**	[1]
30–59 days	3.54%	3.30%		4.11%	3.74%		
60–89 days	37.12%	37.77%		43.46%	42.67%		
> = 90 days	58.54%	58.33%		51.48%	52.89%		
**Other county-level covariates**							
% Uninsured	20.26%	19.13%	**<.0001**	16.76%	15.64%	**<.0001**	
# Dentist/100 k population	54.99	71.03	N/A	59.06	74.70	N/A	[3]
Population/Square Miles	11.08	29.22	N/A	11.08	29.22	N/A	[4]

[1] These are comparison between categorical distributions using Chi-square tests, so there should only be one *p*-value per variable per period

[2] Annual average coverage days per quarter = total coverage days per year (0 ~ 365 or 366) / total covered quarters per year (1–4)

[3] These are weighted average per intervention/control group with no distribution attached, so t-test or Chi-square test would not be applicable (No *p*-value)

[4] For population density, we used 2016 population estimate, so the intervention/control group-level weighted average are the same throughout the study timeframe

#### Annual utilization

Tables 2 and 3 indicates that across all types of services -- use of any dental service and any of eight specific service categories – percentage of users and count of use (if any) per year are similar between PREDICT and control group members. This similarity holds for baseline years and the majority of service types in intervention years, except for count of use of topical fluoride in 2016, as well as percentage of users of any dental, preventive and diagnostic services in 2017.

**Table 2:** Utilization among all covered children and adolescents (Age < = 18) by group and year (2014–2015)

Year	2014 (Baseline Year 1)						2015 (Baseline Year 2)					
Percent vs. Count (if any)	% Use the service			#/svc/yr, if any [Mean (SD)]			% Use the service			#/svc/yr, if any [Mean (SD)]		
Group Comparison	Control	PREDICT	*p*-value	Control	PREDICT	*p*-value	Control	PREDICT	*p*-value	Control	PREDICT	*p*-value
*[Overall Sample Size]*	29,690	59,684	(N/A)	(vary by service types)			32,053	64,329	(N/A)	(vary by service types)		
Any Dental Service	46.14%	45.56%	0.097	8.92 (8.06)	8.84 (7.95)	0.330	43.72%	43.19%	0.118	8.84 (8.03)	8.72 (7.91)	0.176
Any Preventive Service (CDT D1000-D1999)	40.07%	39.49%	0.094	3.91 (3.51)	3.90 (3.47)	0.791	37.66%	37.38%	0.411	3.90 (3.51)	3.85 (3.48)	0.212
Fluoride Varnish (CDT D1206)	24.87%	24.67%	0.503	1.38 (0.78)	1.39 (0.76)	0.411	23.60%	23.12%	0.097	1.37 (0.75)	1.37 (0.76)	0.927
Topical Fluoride, except Varnish (CDT D1208)	10.10%	9.87%	0.265	1.19 (0.43)	1.19 (0.44)	0.863	9.32%	9.31%	0.958	1.20 (0.45)	1.19 (0.45)	0.215
Sealants (CDT D1351)	6.93%	6.51%	0.017	3.10 (1.69)	3.12 (1.81)	0.740	6.07%	6.19%	0.486	3.14 (1.74)	3.14 (1.76)	0.931
Caries Arrest by Silver Diamine Fluoride (CDT D1354)	3.18%	3.12%	0.620	5.16 (7.01)	5.02 (6.85)	0.625	3.07%	3.01%	0.616	5.26 (6.84)	5.06 (6.83)	0.450
Any Diagnostic Service (CDT 0001–0999)	39.57%	39.16%	0.232	3.30 (2.86)	3.26 (2.74)	0.185	37.73%	37.16%	0.088	3.26 (2.67)	3.21 (2.57)	0.081
Any Restorative Service (D2000–2999)	11.67%	11.62%	0.823	2.88 (2.63)	2.81 (2.67)	0.158	10.73%	10.45%	0.179	2.85 (2.66)	2.83 (2.61)	0.721
Any Extraction Services (D7140, D7210, D7250)	3.85%	3.81%	0.780	1.74 (1.29)	1.78 (1.41)	0.411	3.65%	3.53%	0.346	1.87 (1.64)	1.78 (1.40)	0.124

Overall sample sizes are numbers of unique patients in each year

**Table 3:** Utilization among all covered children and adolescents (age < = 18) by group and year (2016–2017)

Year	2016 (Intervention Year 1)						2017 (Intervention Year 2)					
Percent vs. Count (if any)	% Use the service			#/svc/yr, if any [Mean (SD)]			% Use the service			#/svc/yr, if any [Mean (SD)]		
Group Comparison	Control	PREDICT	*p*-value	Control	PREDICT	*p*-value	Control	PREDICT	*p*-value	Control	PREDICT	*p*-value
*[Overall Sample Size]*	32,102	64,232	(N/A)	(vary by service types)			20,395	54,938	(N/A)	(vary by service types)		
Any Dental Service	41.89%	41.63%	0.425	8.69 (8.18)	8.72 (7.90)	0.738	42.86%	43.67%	**0.047**	8.75 (8.00)	8.77 (7.95)	0.779
Any Preventive Service (CDT D1000-D1999)	36.37%	36.11%	0.439	3.81 (3.61)	3.82 (3.48)	0.704	37.03%	37.89%	**0.030**	3.88 (3.54)	3.86 (3.50)	0.607
Fluoride Varnish (CDT D1206)	22.40%	22.21%	0.507	1.37 (0.75)	1.36 (0.73)	0.483	22.79%	23.36%	0.099	1.37 (0.75)	1.37 (0.74)	0.537
Topical Fluoride, except Varnish (CDT D1208)	8.99%	8.91%	0.652	1.17 (0.41)	1.19 (0.44)	**0.038**	9.40%	9.53%	0.599	1.17 (0.42)	1.18 (0.43)	0.392
Sealants (CDT D1351)	5.83%	5.93%	0.540	3.09 (1.75)	3.12 (1.76)	0.620	5.99%	6.35%	0.067	3.11 (1.71)	3.13 (1.74)	0.733
Caries Arrest by Silver Diamine Fluoride (CDT D1354)	2.85%	2.95%	0.384	5.23 (7.58)	5.13 (6.79)	0.732	3.16%	3.02%	0.320	5.12 (6.99)	5.18 (6.94)	0.848
Any Diagnostic Service (CDT 0001–0999)	36.16%	36.09%	0.816	3.24 (2.68)	3.21 (2.66)	0.318	37.00%	37.88%	**0.026**	3.21 (2.57)	3.22 (2.56)	0.753
Any Restorative Service (D2000–2999)	10.01%	10.01%	0.995	2.79 (2.55)	2.84 (2.63)	0.357	10.24%	10.37%	0.602	2.85 (2.65)	2.89 (2.66)	0.593
Any Extraction Services (D7140, D7210, D7250)	3.59%	3.39%	0.120	1.80 (1.37)	1.84 (1.49)	0.472	3.54%	3.63%	0.563	1.73 (1.36)	1.78 (1.37)	0.433

Overall sample sizes are numbers of unique patients in each year

#### Annual average costs of services

Annual cost patterns by service category are essentially the mirror image of annual utilization and described in Table 4. Effectively, costs “monetize” utilization per person by multiplying a constant shadow cost per unit of specific service utilization by count for each specific service.

**Table 4:** Costs of dental services for all covered children (Age < = 18) with at least one use per year

Year	Baseline Period				Intervention Period			
	2014		2015		2016		2017	
Group	Ctrl.	Intv.	Ctrl.	Intv.	Ctrl.	Intv.	Ctrl.	Intv.
***Total Sample Size***	29,690	59,684	32,053	64,232	32,102	64,232	20,395	54,938
***Costs [Mean (S.D.)]***								
Any Use of Dental Services	$213.40 (197.60)	$212.10 (200.90)	$212.10 (205.00)	$208.60 (197.50)	$208.90 (203.60)	$209.20 (200.50)	$208.50 (196.10)	$210.50 (200.20)
Any Preventive Service (CDT D1000-D1999)	$92.18 (70.15)	$92.20 (70.39)	$92.01 (70.54)	$90.77 (69.71)	$89.90 (71.33)	$90.09 (69.69)	$91.12 (69.85)	$90.82 (70.12)
Fluoride Varnish (CDT D1206)	$19.69 (11.16)	$19.82 (10.85)	$19.54 (10.73)	$19.56 (10.81)	$19.51 (10.72)	$19.40 (10.41)	$19.60 (10.65)	$19.49 (10.57)
Topical Fluoride,except Varnish (CDT D1208)	$16.96 (6.11)	$16.99 (6.26)	$17.16 (6.40)	$16.99 (6.43)	$16.64 (5.88)	$16.92 (6.22)	$16.73 (6.01)	$16.87 (6.17)
Sealants (CDT D1351)	$65.92 (35.90)	$66.25 (38.50)	$66.80 (36.86)	$66.71 (37.32)	$65.68 (37.20)	$66.21 (37.47)	$66.10 (36.39)	$66.52 (36.98)
Caries Arrest,by Silver Diamine Fluoride (CDT D1354)	$73.57 (99.98)	$71.65 (97.74)	$75.05 (97.64)	$72.16 (97.50)	$74.64 (108.20)	$73.19 (96.89)	$73.10 (99.68)	$73.98 (99.01)
Any Diagnostic Service (CDT 0001–0999)	$63.40 (38.69)	$62.96 (38.73)	$63.24 (39.02)	$62.57 (38.34)	$63.54 (38.83)	$62.96 (38.25)	$63.03 (37.54)	$63.39 (38.31)
Any Restorative Service (D2000–2999)	$141.20 (156.00)	$137.00 (156.30)	$140.40 (157.70)	$138.40 (153.60)	$137.50 (152.50)	$139.10 (155.00)	$137.50 (152.40)	$141.70 (157.40)
Any Extraction Services (D7140, D7210, D7250)	$167.60 (129.20)	$171.40 (141.30)	$180.20 (165.30)	$170.80 (140.30)	$174.10 (137.00)	$177.50 (149.60)	$167.10 (137.70)	$169.90 (137.10)

(1) *Ctrl* Control, *Intv.* Intervention

(2) Since the distributions of control and intervention group in every year was positively skewed (disproportionately large amount of zeros), two-sample tests are not valid for them

(3) All costs are actual dollar amount in their corresponding years

### Regression results

In this section, only utilization and cost measures significantly affected by PREDICT (with *p* < .05) are discussed, based on ZINB regressions for expected quarterly utilization and two-part models (TPM) for expected quarterly cost. Detailed results, including estimates for effects of PREDICT and covariates for personal and area characteristics are in the Additional file 1.

#### Regression estimates of overall PREDICT effects on specific services

Table 5 summarizes PREDICT average marginal effects on expected use and cost of each dental service type over the entire 8-quarter intervention period. Because the “R_Full” model in Table 5 (columns 3 and 7) takes into account all covariates in Eq. (1), plus the seasonality dummies, we focus on the results of that model. These results complement the next section’s presentation of PREDICT effects by quarter in Figs. 2, 3, and 4.

**Table 5:** Average marginal effect of expected use and cost & covariate[−adjusted baseline trend significance tests

Total Observations = 1,236,967	***Expected Use: ZINB***				***Expected Cost: Two-part Model ($)***			
Dependent Variables: Dental Service Types	Baseline Trend Diff.(1)	Average Marginal Effect			Baseline Trend Diff.(5)	Average Marginal Effect		
		All (2)	R_Full (3)	R_Reduce (4)		All (6)	R_Full (7)	R_Reduce (8)
Any Dental Service (counting all following categories)	−0.023(− 0.056, 0.010)	0.027(− 0.007, 0.061)	0.027(− 0.006, 0.060)	0.024 (− 0.010, 0.058)	−0.573 (−1.309, 0.162)	0.645** (0.012,1.278)	0.636** (0.012,1.259)	0.585* (− 0.067,1.237)
Any Preventive Service (CDT D1000-D1999)	− 0.007 (− 0.017, 0.003)	0.007 (− 0.006, 0.021)	0.007 (− 0.006, 0.020)	0.006 (− 0.007, 0.020)	−0.174 (− 0.402, 0.055)	0.166 (− 0.082, 0.414)	0.163 (− 0.083, 0.409)	0.141 (− 0.110, 0.393)
Any Diagnostic Service (CDT 0001–0999)	− 0.009 (− 0.021, 0.004)	0.009** (0.000, 0.018)	0.009** (0.000, 0.017)	0.008* (− 0.001, 0.017)	− 0.134 (− 0.296, 0.028)	0.142 (− 0.030, 0.313)	0.140 (− 0.030, 0.310)	0.125 (− 0.051, 0.300)
Fluoride Varnish (CDT D1206)	− 0.001 (− 0.006, 0.003)	0.002 (− 0.001, 0.004)	0.004 (− 0.003, 0.011)	0.001 (− 0.003, 0.004)	−0.017 (− 0.077, 0.043)	0.007 (− 0.029, 0.042)	0.006 (− 0.029, 0.042)	0.003 (− 0.031, 0.036)
Topical Fluoride, except Varnish (CDT D1208)	0.000 (− 0.001, 0.001)	0.001** (0.000, 0.002)	0.004 (− 0.015, 0.023)	0.000 (− 0.002, 0.002)	−0.005 (− 0.019, 0.009)	0.011 (− 0.005, 0.027)	0.011 (− 0.005, 0.026)	0.010 (− 0.007, 0.027)
Sealants (CDT D1351)	−0.001 (− 0.004, 0.002)	0.003** (0.000, 0.006)	0.003** (0.000, 0.006)	0.000 (− 0.004, 0.004)	−0.027 (− 0.090, 0.036)	0.064* (− 0.007, 0.135)	0.064* (− 0.006, 0.133)	0.062* (− 0.010, 0.135)
Caries Arrest, by Silver Diamine Fluoride (CDT D1354)	−0.000 (− 0.005, 0.004)	0.002 (− 0.005, 0.009)	0.002 (− 0.005, 0.009)	0.044*** (0.041, 0.048)	−0.011 (− 0.076, 0.054)	0.026 (− 0.058, 0.109)	0.026 (− 0.058, 0.110)	0.024 (− 0.061, 0.109)
Any Restorative Service (D2000–2999)	−0.003 (− 0.008, 0.002)	0.004 (− 0.002, 0.009)	0.003 (− 0.002, 0.009)	0.004 (− 0.002, 0.009)	−0.198 (− 0.489, 0.094)	0.205 (− 0.066, 0.476)	0.203 (− 0.062, 0.467)	0.216 (− 0.060, 0.492)
Any Extraction Services (D7140, D7210, D7250)	−0.000 (− 0.001, 0.001)	0.000 (− 0.001, 0.001)	0.000 (− 0.001, 0.001)	0.000 (− 0.001, 0.001)	−0.033 (− 0.159, 0.093)	0.049 (− 0.073, 0.172)	0.048 (− 0.076, 0.172)	0.037 (− 0.083, 0.157)

1. 95% confidence interval (CI) in parentheses; *** *p* < 0.01, ** *p* < 0.05, * *p* < 0.1

2. All values and their 95% confidence intervals shown here are covariate-adjusted differences between control and PREDICT groups. For baseline period, they are model-adjusted differences between baseline values. For DiD, they are model-adjusted average marginal differences between PREDICT and control group, considering their pre- and post- values

3. Model Setups (all models used “cluster” for county, robust standard errors generated):

a. Baseline differences: utilization/cost = function (secular time [1–8 representing 8 baseline quarters], dummy[control vs. PREDICT], interaction[time * control/PREDICT], seasonality dummies, all usual covariates)

b. DiD – “All”: utilization/cost = function (dummy[pre-period vs. post-period],], dummy[control vs. PREDICT], interaction [dummy[pre-period vs. post-period * control/PREDICT], all usual covariates)

c. DiD – R_Full”: same as “All” model above, but with addition of “Seasonality” dummy variable

d. DiD – R_Reduce: function (dummy[pre-period vs. post-period], dummy[control vs. PREDICT], interaction[pre/post* control/PREDICT], seasonality dummies), but no other covariates included in this model

4. Setting of the Difference-in-Differences Average Marginal Effects:

a. Use: [Use (PREDICT, post-intervention period 2016–2017) – Use (PREDICT, pre-intervention period 2014–2015)] – [Use (CONTROL, post-intervention period 2016–2017) – Use (CONTROL, pre-intervention period 2014–2015)],

b. Cost: same structure as “Use”, replace all “use” items by their corresponding “cost” items

5. AME on Expected Use illustrate the change in expected use as an impact from the PREDICT program

6. AME on Expected Cost illustrate the change in the expected cost as an impact from the PREDICT program

7. The ZINB models on “Use of Fluoride Varnish”, “Use of Topical Fluoride”, and “Use of Caries Arrest” did not achieve convergence, so the coefficient on their effects are not statistically reliable

8. Significance levels were calculated using the original un-rounded numbers, so the calculation of the face value (4-digit point rounded) in the table may not show significance levels as indicated due to rounding omissions

Three statistically significant (*p* < .05) average marginal effects of PREDICT per quarter per child were observed in the R_Full model (our preferred specification) (1) an increase of $0.64 in total expected cost for all services received; (2) an increase of 0.009 units in expected use of diagnostic services; (3) increase of 0.003 units and $0.06 in expected use and expected cost of sealants, respectively.

#### Regression estimates of PREDICT effects by quarter on specific services

Figure 2 (Panels A thru F) displays estimated average marginal effects (AME) by quarter of PREDICT on expected utilization and expected cost of dental services overall, diagnostic, and preventive services. The AMEs estimate the combined effect of PREDICT on each dependent measure, integrating impact on probability of any utilization (or cost) with impact on level of utilization (or cost), given positive utilization (or cost). Except for a slight decline (< $0.10; *p* < .05) of expected quarterly diagnostic service costs in intervention quarter 2, none of these quarterly effects was statistically significant. In contrast, use of diagnostic services over the entire intervention period was significantly higher (Table 5) in the PREDICT group which is consistent with the mostly positive point estimates by quarter in Fig. 3.

#### DID regression model results for expected use of any services

Table 6 explores the determinants of two utilization measures: (1) probability of use of any service and (2) number of services given any use (Panel A). Use of any service was an important component of incentive metrics used in the compensation model. This sub-analysis offers a more detailed examination of determinants of overall access to dental services (any use).

**Table 6:** Use and cost of any dental services: difference-in-differences analyses

***% Change Rate [exp(b)-1]***	Any Dental: Diff-in-Diff (2016Q1 - 2017Q4 vs. Baseline All)			
Total # of Patient Quarters = 1,236,967	Utilization: ZINB model		Cost: two-part model	
Covariates	Inflate (0 vs. 1)	Count	Logit (1 vs. 0)	GLM
**Age (ref: 0 to 5)**				
6 to 12	−0.218*** (0.008)	− 0.003 (0.006)	0.267*** (0.013)	− 0.006 (0.005)
13 to 18	−0.112*** (0.008)	− 0.052*** (0.008)	0.110*** (0.011)	− 0.028*** (0.004)
**Gender (ref: Female)**				
Male	−0.002 (0.007)	− 0.006* (0.003)	0.001 (0.007)	−0.003 (0.003)
**Race/Ethnicity (ref: White)**				
Hispanic Non-White	−0.027*** (0.008)	−0.007* (0.004)	0.026*** (0.008)	−0.010*** (0.003)
Other	0.110*** (0.014)	0.006 (0.008)	−0.096*** (0.012)	0.008* (0.004)
**Coverage Days (ref: < 30 days)**				
30–59 days	−0.051*** (0.018)	− 0.038* (0.019)	0.045** (0.020)	−0.037*** (0.013)
60–89 days	− 0.064*** (0.019)	−0.035*** (0.013)	0.062*** (0.023)	−0.028* (0.014)
> = 90 days	− 0.102*** (0.016)	− 0.033** (0.013)	0.105*** (0.020)	− 0.030*** (0.011)
**Other county-level covariates**				
Uninsured %Point (1–100)	−0.005 (0.003)	0.002 (0.002)	0.005 (0.003)	0.000 (0.001)
# Dentist/100 k population	0.000 (0.001)	−0.000 (0.000)	−0.000 (0.001)	− 0.000 (0.000)
Population/Square Miles	0.000 (0.000)	0.000** (0.000)	−0.000 (0.000)	0.000*** (0.000)
**PREDICT (ref: Control)**	0.007 (0.019)	−0.014** (0.006)	−0.010 (0.018)	− 0.012* (0.006)
**Post-intervention Quarters (ref: baseline all)**				
2016Q1	0.076*** (0.009)	−0.010 (0.015)	−0.069*** (0.008)	− 0.015 (0.016)
2016Q2	0.040** (0.020)	−0.001 (0.014)	−0.038** (0.018)	− 0.004 (0.012)
2016Q3	0.067*** (0.023)	−0.004 (0.019)	−0.062*** (0.021)	− 0.009 (0.014)
2016Q4	0.072*** (0.017)	0.002 (0.016)	−0.065*** (0.017)	0.008 (0.014)
2017Q1	0.075** (0.031)	0.038** (0.018)	−0.065** (0.026)	0.020 (0.014)
2017Q2	0.054* (0.029)	0.002 (0.022)	−0.050* (0.025)	−0.003 (0.018)
2017Q3	0.046 (0.040)	−0.002 (0.016)	−0.041 (0.036)	− 0.017 (0.015)
2017Q4	0.058 (0.039)	0.019 (0.017)	−0.048 (0.035)	−0.000 (0.011)
**Interaction Effect (ref: Control at baseline all)**				
PREDICT/2016Q1	−0.030** (0.015)	0.011 (0.019)	0.030* (0.016)	0.015 (0.020)
PREDICT/2016Q2	0.011 (0.019)	0.011 (0.015)	−0.008 (0.019)	0.015 (0.014)
PREDICT/2016Q3	−0.009 (0.027)	0.028 (0.020)	0.015 (0.029)	0.027* (0.015)
PREDICT/2016Q4	−0.021 (0.017)	0.005 (0.023)	0.022 (0.019)	−0.011 (0.018)
PREDICT/2017Q1	−0.046** (0.019)	−0.014 (0.015)	0.047** (0.022)	−0.003 (0.014)
PREDICT/2017Q2	−0.003 (0.019)	0.022 (0.023)	0.007 (0.019)	0.011 (0.020)
PREDICT/2017Q3	−0.004 (0.025)	0.053*** (0.019)	0.010 (0.026)	0.053*** (0.017)
PREDICT/2017Q4	−0.021 (0.019)	−0.014 (0.015)	0.017 (0.020)	−0.002 (0.010)
**Constant**	3.963*** (0.378)	4.432*** (0.267)	−0.819*** (0.013)	136.064*** (4.135)

Robust standard errors in parentheses; *** *p* < 0.01, ** *p* < 0.05, * *p* < 0.1; Clustering on “county” to adjust for within-county homoskedasticity; Alpha test ZINB vs. ZIP = − 0.441*** (0.005) [Significant means ZINB is preferred over ZIP]

Panel A reveals that, relative to ages 0–5 (i.e., pre-school: the reference category), children ages 6–12 were the most likely to receive any care (24.4 percentage points more than pre-school), followed by teenagers. Relative to white children and adolescents, Hispanic non-white adolescents were more likely to access some care (by 2.7 percentage points), and other non-White racial and ethnic groups were less likely (by 10.4 percentage points).

More days of coverage within quarter increased probability of utilization. PREDICT effects on probability of utilization were positive in all but two intervention quarters, but generally small and statistically insignificant -- except for quarter 1 (3.0 percentage point increase) and quarter 5 (4.7 percentage point increase). Population density had a statistically significant, but very small, impact on level of utilization of any services. Neither percent of population medically uninsured nor dentist-to-population ratio significantly affected children’s utilization or total costs.

Figure 3 (Panels A thru F) illustrates average marginal effects of PREDICT by quarter on expected use and cost of fluoride varnish, preventive silver diamine fluoride without varnish, and sealants. The only statistically significant PREDICT effect is a small increase in intervention quarter 5 in expected utilization of sealants (.01 sealant per child) and expected sealant cost ($2 per child). These quarterly impacts are consistent with results in Table 5 over the entire intervention period: a small average increase in expected use and costs of sealants of .003 sealants and $0.064 per child per quarter.

Figure 4 depicts PREDICT effects for caries arrest, restorative services, and extractions. Only one average marginal effect is statistically significant: a decline of approximately $2 per person in expected cost of caries arrest services in quarter 8. Given the close relationship between expected cost and expected use, failure of the ZINB count model for caries arrest to converge casts some doubt on validity of this estimate.

## Discussion

The principal hypothesis of this study – that primary dental care (preventive and diagnostic) would increase significantly over time in PREDICT counties relative to controls – was somewhat supported in our findings (see Table 5). There were small, but statistically significant increases in differential use of diagnostic services, topical fluoride, and sealants. However, there was no decline in total dental costs. Instead children and adolescents in PREDICT counties experienced a small, but statistically significant rise in total costs. While there was no clear tradeoff between primary care and more expensive procedures, neither did restorative and extraction procedures increase significantly.

The incentive payment dollars earned by participating providers in the PREDICT (test) counties were intentionally not included in the costs measured in this study. By using allowed payments by Medicaid for each service as our “cost” metric, the authors chose a “shadow cost” to reflect the mutually acceptable amount that Medicaid was willing to pay for each service and that the providers would accept to cover the provider’s cost of delivering that service. The study was not intended to assess the cost- effectiveness of the incentive program. Such a study would have included Medicaid’s direct cost of the incentive payments, as well as the costs to Medicaid and participating providers alike of implementing the incentive program. The authors explicitly acknowledge that such a study (not this one) might demonstrate that the direct cost of incentive payments plus any program implementation costs exceeded any dental service cost savings realized as a result of PREDICT.

### Limitations

The relatively brief observation period (2 years) to identify clinically and economically meaningful and statistically significant effects of PREDICT might account for the modest impacts described in this paper. Changes in care delivery and provider incentives constituted major innovations relative to prevailing arrangements, but previous research has established that full impacts of major innovation generally require several years to appear [23, 24]. Moreover, the hub and spoke model was not fully implemented because not all school sites were covered, and hub practices continued to see a significant number of children and adolescents who potentially could have been seen at community sites.

The small number of counties in the randomization might mean that imbalances in provider, patient, and area characteristics remain between PREDICT and control counties. To confound our estimates of intervention effects, however, these potentially omitted variables would have to be correlated with our utilization and cost variables. Our tests of parallel baseline trend suggest that such potential confounding is not present in this study. Furthermore, the cluster-randomized design does rule out systematic selection bias The authors therefore argue that our regression covariates have captured salient differences between the intervention and control groups and have effectively eliminated potential omitted variable bias in the estimates.

Spillover effects on care models and compensation between PREDICT and control counties cannot be ruled out, especially given the presence of ADS in both sets of counties and inherent sharing of best practices among providers and practice administrators in a DCO. Such spillover effects likely reduced observed differences.

### Summary

On the whole, our findings, combined with the foregoing caveats, imply that these estimates of certain modest PREDICT effects do not definitively demonstrate causation or substantial change attributable to the intervention. That said, our formal statistical tests suggest that the modest PREDICT effects observed in this study are not tainted by bias and are plausibly attributable to this intervention. Our findings illustrate the challenge in bending the cost curve and achieving improved outcomes.

Only a few categories of utilization and cost for children and adolescents enrolled in Medicaid were significantly influenced by PREDICT. However, a close look at this study’s findings suggests favorable developments within PREDICT counties in enhancing preventive and diagnostic procedures, while simultaneously holding the line on expensive restorative and extraction procedures. In that sense, efforts by this DCO may be bearing fruit, even if certain impacts were not unambiguously attributable to PREDICT per se. This study adds significantly to a sparse literature on dental care QI and innovations of this type.

## Supplementary Information

**Additional file 1: Appendix 1.** Parallel Trend Tests. To assess the validity of our DID estimates of PREDICT effects, we briefly summarize the results of parallel trend tests for specific services. Our parallel trend tests for each service type examine whether the regression-adjusted differences in baseline values of utilization and cost between the PREDICT and control group are statistically significant, examining each of the eight baseline quarters in 2014 and 2015. **Appendix 2.** Estimated Difference-in-Difference (DID) Regression Models.

## Data Availability

The data that support the findings of this study are available from the Dr. Milgrom upon reasonable request.
