# Supplementary material for: Impacts of innovation in dental care delivery and payment in Medicaid managed care for children and adolescents
Source: BMC Health Serv Res. 2021 Jun 8;21:565. doi: 10.1186/s12913-021-06549-3 (PMC8188686; doi:10.1186/s12913-021-06549-3)
Supplement: Supplementary file 1 — Additional file 1: Appendix 1. Parallel Trend Tests. To assess the validity of our DID estimates of PREDICT effects, we briefly summarize the results of parallel trend tests for specific services. Our parallel trend tests for each service type examine whether the regression-adjusted differences in baseline values of utilization and cost between the PREDICT and control group are statistically significant, examining each of the eight baseline quarters in 2014 and 2015. Appendix 2. Estimated Difference-in-Difference (DID) Regression Models. [file 12913_2021_6549_MOESM1_ESM.docx]

**Impacts of Innovation in Dental Care Delivery and Payment in Medicaid Managed Care for Children and Adolescents**

Douglas A. Conrad^1^, Peter Milgrom^2 *^, Yuxian Du^3^, Joana Cunha-Cruz^4^, Sharity Ludwig^5^, and R. Mike Shirtcliff DMD^6^

**Appendices: Supplementary Materials**

**Appendix (1): Parallel Trend Tests.** To assess the validity of our DID estimates of PREDICT effects, we briefly summarize the results of parallel trend tests for specific services. Our parallel trend tests for each service type examine whether the regression-adjusted differences in baseline values of utilization and cost between the PREDICT and control group are statistically significant , examining each of the eight baseline quarters in 2014 and 2015.

Figures FA1, FA2, and FA3 in the Supplementary Appendix present results of our parallel trend tests for the nine expected quarterly utilization and corresponding quarterly expected cost variables. With the exception of expected utilization and cost of preventive services in quarter 4 and expected use of diagnostic services in quarter 8 , Figure FA1 illustrates that baseline quarterly expected cost and utilization are not significantly different between the PREDICT and control group in any quarter for dental services overall, diagnostic services, or preventive services.

**<Insert Figure FA1 here>**

Figure FA2 shows that one cannot reject the null hypothesis of parallel trends for expected cost and utilization of fluoride varnish, silver diamine topical fluoride without varnish, or sealants.

**<Insert Figure FA2 here>**

Finally, in Figure FA3 -- similar to results for the other six service types - the regression-adjusted baseline quarterly expected use and cost differences are statistically insignificant for expected cost of caries arrest services, and also for utilization and cost of restorations and extractions – with the singular exception of extractions in baseline quarter 6. The regression model for baseline caries arrest services utilization did not converge, so we cannot rely on those results to test for parallel trends of caries arrest services in this quarterly specification..

**<Insert Figure FA3 here>**

Given these results, we conclude that the assumption of parallel trends is generally, but not uniformly, supported in this test of quarter-specific, covariate-adjusted baseline differences. However, as reported in the main body of the paper and displayed in Table 4 columns (1) and (5), the parallel baseline trends assumption is uniformly confirmed in the model that adjusts for general seasonal (by quarter) variation and tests for linear baseline trend differences between the PREDICT and control group. Since the latter provides a more powerful test, we base our conclusions regarding parallel baseline trends on that model.

**Figures FA1, FA2, and FA3 follow:**

**Covariate-Adjusted Baseline Trend Differences between PREDICT**

**and the Control Group by Quarter by Service Type**

These Figures are on the following pages in order:

**Figure FA1: for Service Types of Any Dental, Diagnostic, and Preventive**

**Figure FA2: for Service Types of Fluoride Varnish, Topical Fluoride, and Sealants**

**Figure FA3: for Service Types of Caries Arrest, Restorative, and Extractions**

**Figure FA1. Baseline Differences in ‘Covariate-Adjusted’ Values: Any, Diagnostic, and Preventive (PREDICT vs. Control)**

| Panel A. Expected Cost of Any Dental  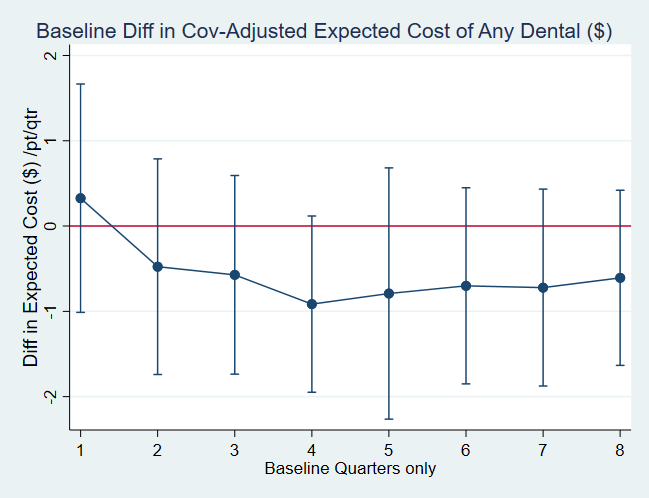 | Panel B: Expected Count of Any Dental  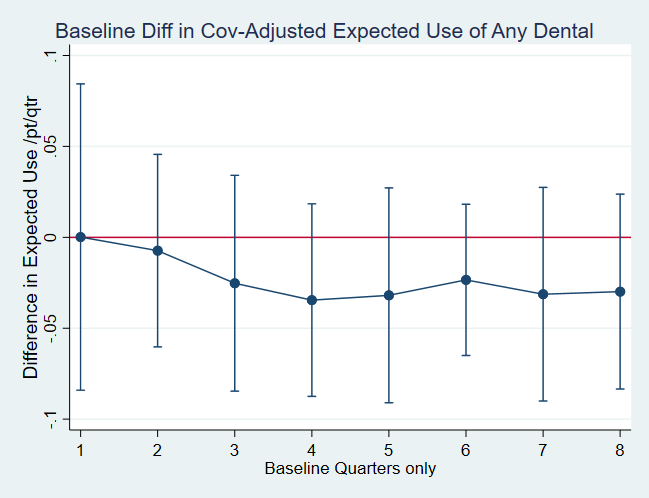 |
| --- | --- |
| Panel C: Expected Cost of Any Diagnostic  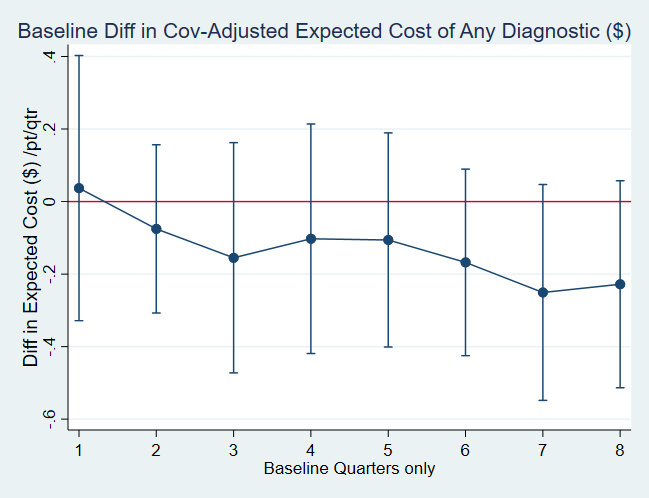 | Panel D: Expected Count of Any Diagnostic  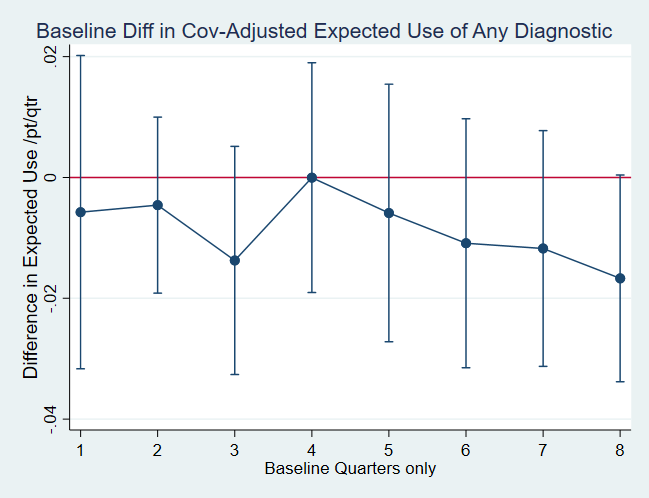 |
| Panel E: Expected Cost of Any Preventive  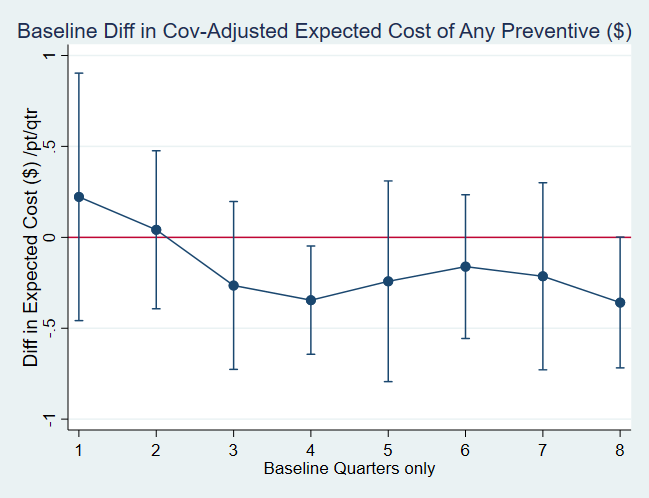 | Panel F: Expected Count of Any Preventive  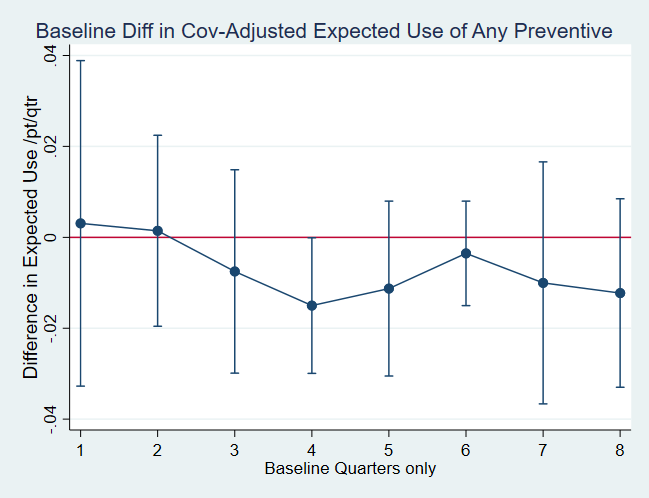 |

The hashmarks at each end represent the upper and lower bounds of the 95% confidence interval.

### **Figure FA2. Baseline Differences in ‘Covariate-Adjusted’ Values: Fluoride Varnish, Topical Fluoride, and Sealants (PREDICT vs. Control)**

| Panel A. Expected Cost of Fluoride Varnish  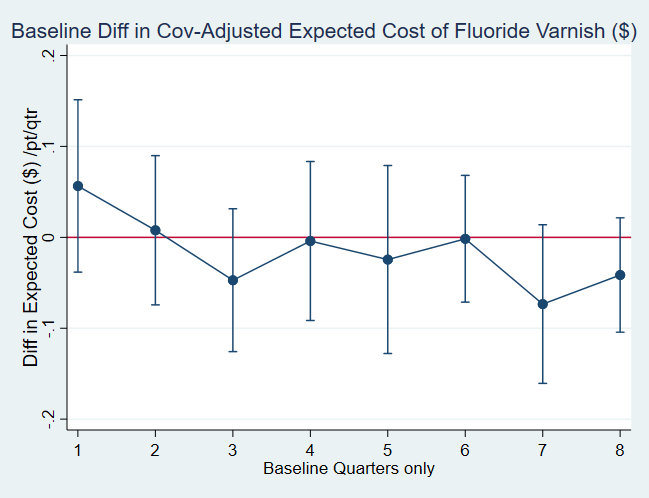 | Panel B. Expected Count of Fluoride Varnish  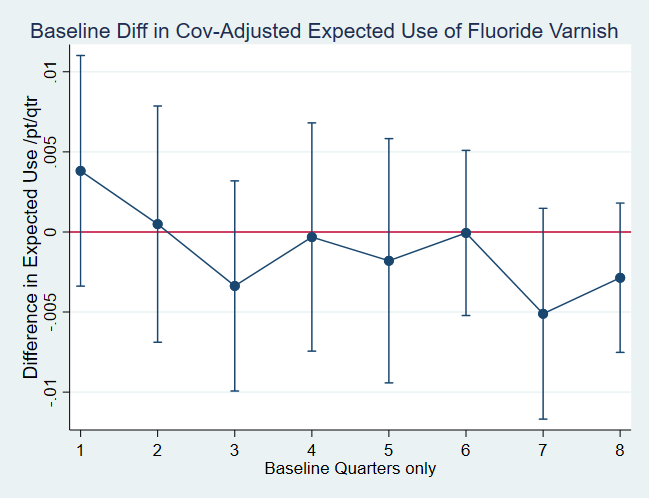 |
| --- | --- |
| Panel C. Expected Cost of Topical Fluoride  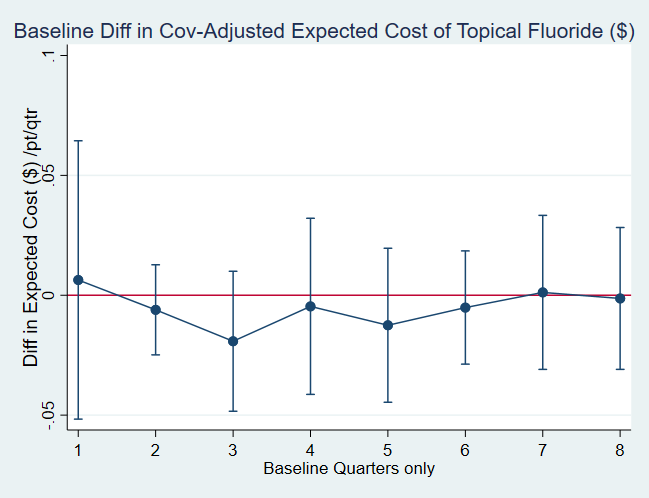 | Panel D. Expected Count of Topical Fluoride  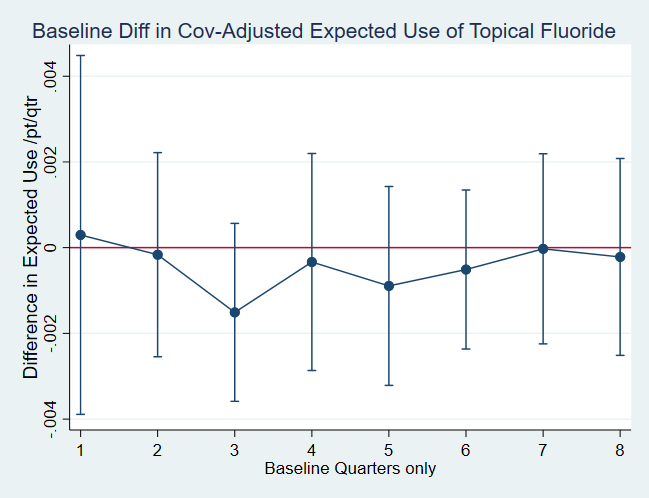 |
| Panel E. Expected Cost of Sealants  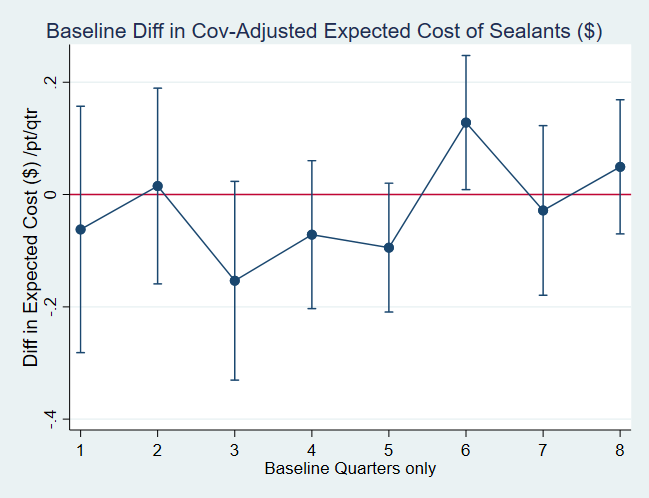 | Panel F. Expected Count of Sealants  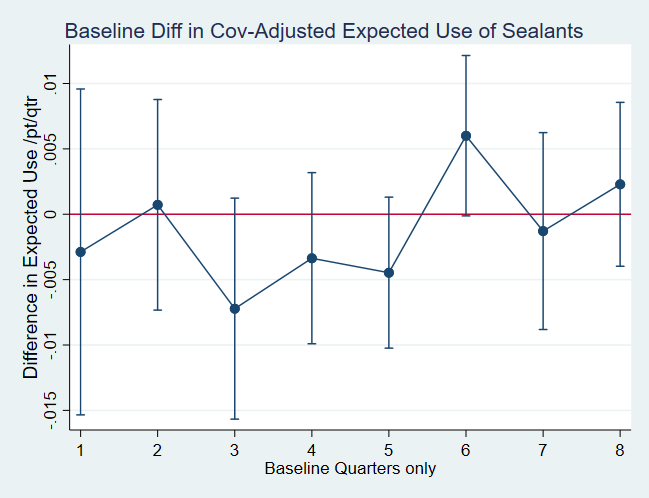 |

**Note: The models on “Use of Fluoride Varnish” and “Use of Topical Fluoride” did not achieve convergence. The hashmarks at each end represent the upper and lower bounds of the 95% confidence interval.**

### **Figure FA3. Baseline Differences in ‘Covariate-Adjusted’ Values: Caries Arrest, Restorative, and Extractions (PREDICT vs. Control)**

| Panel A. Expected Cost of Caries Arrest  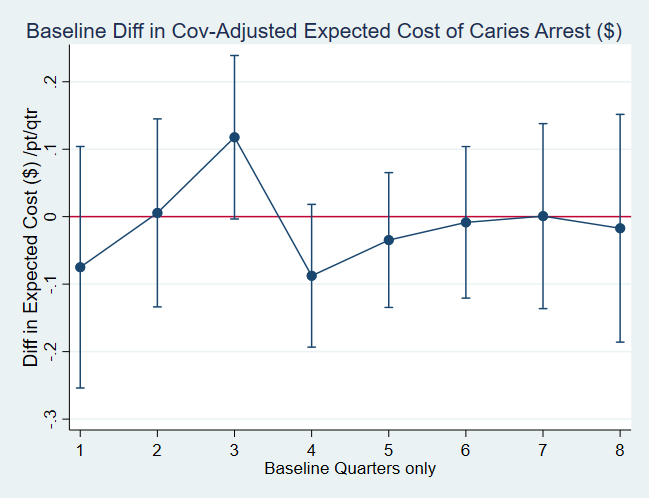 | Panel B. Expected Count of Caries Arrest  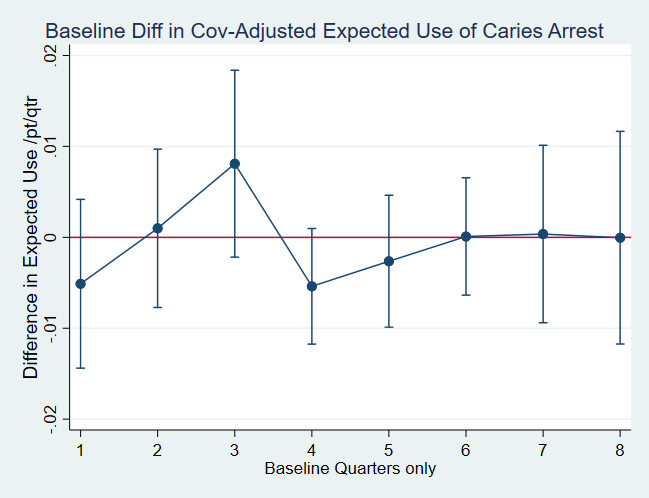 |
| --- | --- |
| Panel C. Expected Cost of Restorative  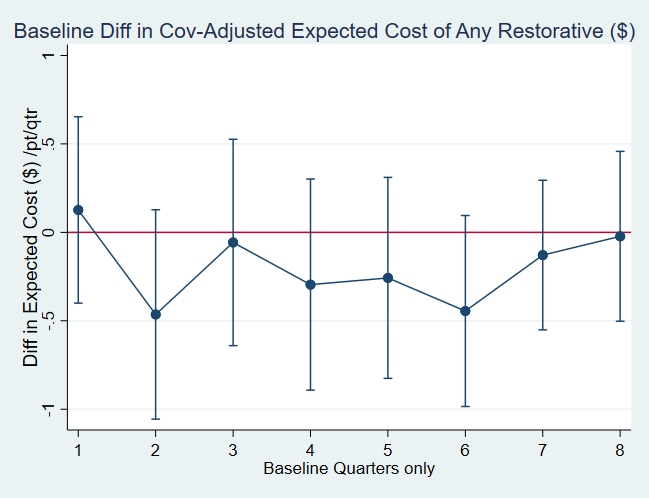 | Panel D. Expected Count of Restorative  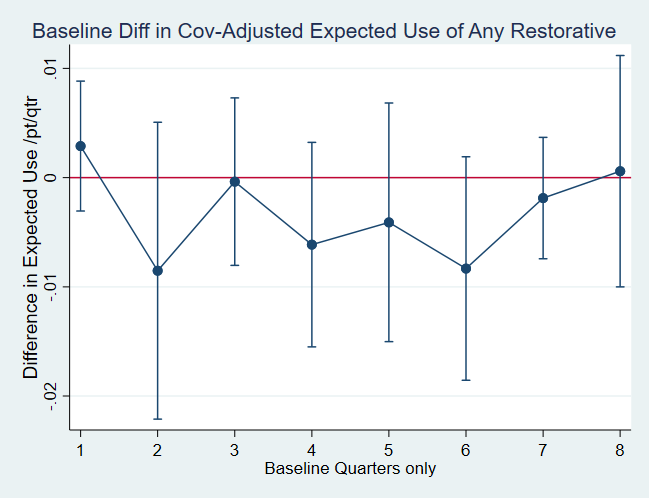 |
| Panel E. Expected Cost of Extractions  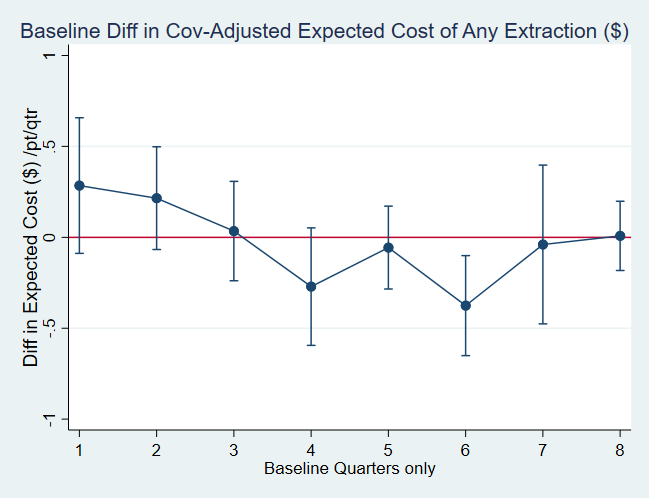 | Panel F. Expected Count of Extractions  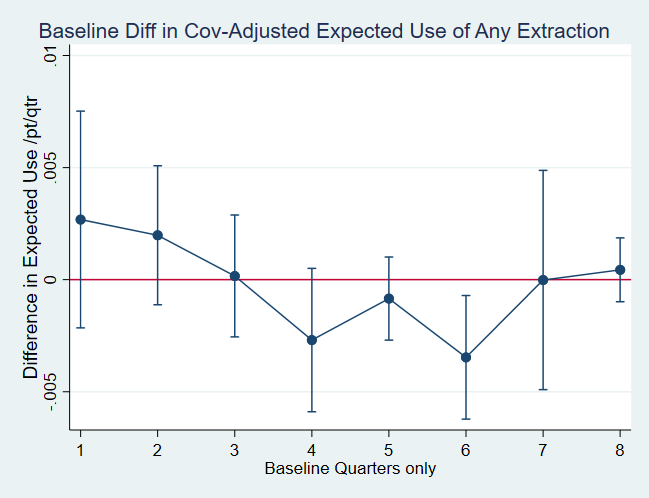 |

**Note: The model on “use of caries arrest” did not achieve convergence. The hashmarks at each end represent the upper and lower bounds of the 95% confidence interval.**

**Appendix (2): Estimated Difference-in-Difference (DID) Regression Models.**

**Table TA1: DID Estimates for Use and Cost of Preventive Services**

**Table TA2: DID Estimates for Use and Cost of Diagnostic Services**

**Table TA3: DID Analyses for Use and Cost of Sealants**

**Table TA4: DID Analyses for Use and Cost of Restorative Services**

**(The tables appear in this order starting next page).**

### ***Table TA1. Use and Cost of Any Preventive Services: Difference-in-Differences Analyses***

| ***% Change Rate [exp(b)-1]*** | **Any Preventive: Diff-in-Diff (2016Q1 - 2017Q4 vs. Baseline All)** | | | |
| --- | --- | --- | --- | --- |
| Total # of Patient Quarters = 1,236,967 | **Utilization: ZINB model** | | **Cost: two-part model** | |
| Covariates | Inflate (0 vs. 1) | Count | Logit (1 vs. 0) | GLM |
| **Age (ref: 0 to 5)** |  |  |  |  |
| 6 to 12 | -0.179*** (0.010) | 0.033*** (0.009) | 0.221*** (0.015) | 0.042*** (0.005) |
| 13 to 18 | -0.024 (0.018) | 0.015*** (0.005) | 0.029 (0.018) | 0.052*** (0.003) |
| **Gender (ref: Female)** |  |  |  |  |
| Male | -0.006 (0.006) | -0.009* (0.005) | 0.002 (0.005) | -0.002 (0.003) |
| **Race/Ethnicity (ref: White)** |  |  |  |  |
| Hispanic Non-White | -0.024*** (0.008) | 0.002 (0.007) | 0.024*** (0.008) | 0.004 (0.005) |
| Other | 0.107*** (0.018) | 0.006 (0.012) | -0.091*** (0.015) | 0.001 (0.007) |
| **Coverage Days (ref: <30 days)** |  |  |  |  |
| 30-59 days | -0.036** (0.017) | -0.023 (0.020) | 0.027 (0.021) | -0.019 (0.012) |
| 60-89 days | -0.055*** (0.020) | -0.019 (0.017) | 0.049** (0.022) | -0.012 (0.011) |
| >= 90 days | -0.088*** (0.014) | -0.009 (0.015) | 0.088*** (0.018) | -0.006 (0.008) |
| **Other county-level covariates** |  |  |  |  |
| Uninsured %Point (1-100) | -0.007** (0.003) | 0.000 (0.002) | 0.006** (0.003) | -0.000 (0.001) |
| # Dentist/100k population | 0.000 (0.000) | 0.000 (0.000) | -0.000 (0.000) | 0.000 (0.000) |
| Population/Square Miles | -0.000 (0.000) | -0.000 (0.000) | 0.000 (0.000) | -0.000** (0.000) |
| **PREDICT (ref: Control)** | 0.007 (0.014) | -0.009* (0.006) | -0.010 (0.014) | -0.005 (0.004) |
| **Post-intervention Quarters (ref: baseline all)** | |  |  |  |
| 2016Q1 | 0.064*** (0.016) | -0.018 (0.015) | -0.063*** (0.014) | -0.011 (0.008) |
| 2016Q2 | 0.028 (0.021) | -0.007 (0.018) | -0.028 (0.017) | -0.004 (0.007) |
| 2016Q3 | 0.053* (0.029) | -0.012 (0.024) | -0.052** (0.024) | -0.015 (0.012) |
| 2016Q4 | 0.057*** (0.017) | -0.019 (0.023) | -0.057*** (0.019) | -0.009 (0.015) |
| 2017Q1 | 0.037 (0.030) | 0.011 (0.022) | -0.030 (0.025) | -0.005 (0.011) |
| 2017Q2 | 0.009 (0.024) | -0.027 (0.020) | -0.018 (0.024) | -0.014 (0.013) |
| 2017Q3 | 0.044 (0.037) | 0.002 (0.016) | -0.040 (0.034) | -0.006 (0.012) |
| 2017Q4 | 0.041 (0.041) | 0.009 (0.023) | -0.034 (0.036) | -0.010 (0.012) |
| **Interaction Effect (ref: Control at baseline all)** | |  |  |  |
| PREDICT/2016Q1 | -0.028 (0.020) | 0.003 (0.026) | 0.029 (0.021) | -0.004 (0.014) |
| PREDICT/2016Q2 | 0.013 (0.022) | -0.000 (0.020) | -0.012 (0.018) | -0.001 (0.008) |
| PREDICT/2016Q3 | -0.029 (0.030) | 0.003 (0.024) | 0.029 (0.030) | 0.009 (0.012) |
| PREDICT/2016Q4 | -0.011 (0.020) | 0.023 (0.033) | 0.018 (0.021) | 0.004 (0.020) |
| PREDICT/2017Q1 | -0.028 (0.022) | -0.002 (0.025) | 0.027 (0.021) | 0.005 (0.013) |
| PREDICT/2017Q2 | 0.009 (0.020) | 0.015 (0.025) | -0.003 (0.019) | -0.002 (0.014) |
| PREDICT/2017Q3 | -0.036 (0.025) | 0.008 (0.017) | 0.039 (0.028) | 0.006 (0.011) |
| PREDICT/2017Q4 | -0.038 (0.027) | -0.031 (0.021) | 0.025 (0.026) | -0.007 (0.012) |
| **Constant** | 4.458*** (0.406) | 1.209*** (0.112) | -0.861*** (0.009) | 63.819*** (1.737) |

Notes for Table A1: Robust standard errors in parentheses; *** p<0.01, ** p<0.05, * p<0.1; Clustering on "county" to adjust for within-county homoskedasticity; Alpha test ZINB vs. ZIP = -0.566*** (0.009) [Significant means ZINB is preferred over ZIP]

### ***Table TA2. Use and Cost of Any Diagnostic Services: Difference-in-Differences Analyses***

| ***% Change Rate [exp(b)-1]*** | **Any Diagnostic: Diff-in-Diff (2016Q1 - 2017Q4 vs. Baseline All)** | | | |
| --- | --- | --- | --- | --- |
| Total # of Patient Quarters = 1,236,967 | **Utilization: ZINB model** | | **Cost: two-part model** | |
| Covariates | Inflate (0 vs. 1) | Count | Logit (1 vs. 0) | GLM |
| **Age (ref: 0 to 5)** |  |  |  |  |
| 6 to 12 | -0.122*** (0.011) | 0.089*** (0.008) | 0.171*** (0.012) | 0.021*** (0.003) |
| 13 to 18 | 0.039*** (0.014) | 0.105*** (0.008) | 0.006 (0.013) | 0.050*** (0.005) |
| **Gender (ref: Female)** |  |  |  |  |
| Male | 0.002 (0.009) | -0.000 (0.006) | -0.002 (0.007) | 0.003 (0.002) |
| **Race/Ethnicity (ref: White)** |  |  |  |  |
| Hispanic Non-White | -0.015** (0.007) | 0.001 (0.011) | 0.015*** (0.005) | 0.000 (0.005) |
| Other | 0.078*** (0.013) | -0.018** (0.007) | -0.075*** (0.010) | 0.004 (0.003) |
| **Coverage Days (ref: <30 days)** |  |  |  |  |
| 30-59 days | -0.049** (0.024) | -0.024 (0.023) | 0.038* (0.023) | -0.027*** (0.010) |
| 60-89 days | -0.054** (0.022) | -0.028* (0.016) | 0.041* (0.025) | -0.017* (0.009) |
| >= 90 days | -0.090*** (0.016) | -0.035** (0.016) | 0.076*** (0.021) | -0.031*** (0.008) |
| **Other county-level covariates** |  |  |  |  |
| Uninsured %Point (1-100) | -0.004 (0.002) | 0.004** (0.002) | 0.005** (0.002) | 0.001 (0.001) |
| # Dentist/100k population | 0.000 (0.000) | 0.000 (0.000) | -0.000 (0.000) | 0.000* (0.000) |
| Population/Square Miles | -0.000 (0.000) | -0.000 (0.000) | -0.000 (0.000) | -0.000* (0.000) |
| **PREDICT (ref: Control)** | 0.009 (0.013) | -0.016* (0.008) | -0.015 (0.014) | -0.007*** (0.003) |
| **Post-intervention Quarters (ref: baseline all)** | |  |  |  |
| 2016Q1 | 0.040*** (0.014) | -0.023 (0.017) | -0.046*** (0.010) | -0.003 (0.007) |
| 2016Q2 | 0.020 (0.021) | -0.001 (0.009) | -0.019 (0.019) | 0.022*** (0.006) |
| 2016Q3 | 0.029 (0.023) | -0.028 (0.023) | -0.038 (0.023) | -0.008 (0.013) |
| 2016Q4 | 0.051*** (0.020) | -0.007 (0.011) | -0.049*** (0.018) | 0.011 (0.008) |
| 2017Q1 | 0.045* (0.026) | -0.001 (0.028) | -0.041* (0.022) | -0.004 (0.011) |
| 2017Q2 | 0.042 (0.026) | -0.007 (0.027) | -0.041* (0.022) | 0.008 (0.013) |
| 2017Q3 | 0.013 (0.036) | -0.017 (0.017) | -0.019 (0.033) | -0.000 (0.015) |
| 2017Q4 | 0.020 (0.032) | -0.014 (0.020) | -0.024 (0.031) | 0.015* (0.008) |
| **Interaction Effect (ref: Control at baseline all)** | |  |  |  |
| PREDICT/2016Q1 | -0.010 (0.019) | 0.023 (0.027) | 0.019 (0.015) | 0.014 (0.011) |
| PREDICT/2016Q2 | 0.003 (0.023) | -0.008 (0.015) | -0.006 (0.020) | -0.017** (0.007) |
| PREDICT/2016Q3 | 0.009 (0.029) | 0.050* (0.028) | 0.012 (0.029) | 0.026* (0.016) |
| PREDICT/2016Q4 | -0.024 (0.020) | -0.009 (0.017) | 0.020 (0.020) | -0.008 (0.010) |
| PREDICT/2017Q1 | -0.043* (0.023) | -0.003 (0.025) | 0.041** (0.020) | 0.012 (0.011) |
| PREDICT/2017Q2 | -0.016 (0.027) | 0.023 (0.024) | 0.025 (0.025) | 0.004 (0.012) |
| PREDICT/2017Q3 | 0.000 (0.030) | 0.058*** (0.023) | 0.023 (0.027) | 0.028 (0.019) |
| PREDICT/2017Q4 | -0.013 (0.021) | 0.006 (0.021) | 0.015 (0.021) | -0.008 (0.009) |
| **Constant** | 3.897*** (0.318) | 0.664*** (0.083) | -0.865*** (0.008) | 47.153*** (0.936) |

Notes for Table A2: Robust standard errors in parentheses; *** p<0.01, ** p<0.05, * p<0.1; Clustering on "county" to adjust for within-county homoskedasticity; Alpha test ZINB vs. ZIP = -0.494*** (0.014) [Significant means ZINB is preferred over ZIP]

### ***Table TA3. Use and Cost of Sealants: Difference-in-Differences Analyses***

| ***% Change Rate [exp(b)-1]*** | **Sealants: Diff-in-Diff (2016Q1 - 2017Q4 vs. Baseline All)** | | | |
| --- | --- | --- | --- | --- |
| Total # of Patient Quarters = 1,236,967 | **Utilization: ZINB model** | | **Cost: two-part model** | |
| Covariates | Inflate (0 vs. 1) | Count | Logit (1 vs. 0) | GLM |
| **Age (ref: 0 to 5)** |  |  |  |  |
| 6 to 12 | -0.416*** (0.010) | -0.039*** (0.009) | 0.695*** (0.028) | -0.031*** (0.007) |
| 13 to 18 | -0.201*** (0.029) | 0.012 (0.016) | 0.254*** (0.041) | 0.009 (0.012) |
| **Gender (ref: Female)** |  |  |  |  |
| Male | 0.003 (0.010) | -0.006 (0.011) | -0.004 (0.010) | -0.005 (0.009) |
| **Race/Ethnicity (ref: White)** |  |  |  |  |
| Hispanic Non-White | -0.016 (0.018) | -0.000 (0.011) | 0.016 (0.018) | -0.000 (0.008) |
| Other | 0.089*** (0.023) | 0.042*** (0.012) | -0.074*** (0.019) | 0.034*** (0.009) |
| **Coverage Days (ref: <30 days)** |  |  |  |  |
| 30-59 days | 0.008 (0.102) | -0.002 (0.030) | -0.009 (0.102) | -0.002 (0.024) |
| 60-89 days | -0.019 (0.074) | -0.002 (0.041) | 0.018 (0.074) | -0.002 (0.033) |
| >= 90 days | -0.050 (0.070) | -0.004 (0.022) | 0.052 (0.077) | -0.004 (0.018) |
| **Other county-level covariates** |  |  |  |  |
| Uninsured %Point (1-100) | -0.009* (0.005) | -0.006* (0.003) | 0.008 (0.006) | -0.005* (0.002) |
| # Dentist/100k population | 0.001 (0.001) | 0.001 (0.001) | -0.001 (0.001) | 0.001 (0.001) |
| Population/Square Miles | -0.001 (0.001) | -0.000*** (0.000) | 0.000 (0.001) | -0.000*** (0.000) |
| **PREDICT (ref: Control)** | 0.020 (0.023) | 0.000 (0.018) | -0.020 (0.021) | -0.000 (0.014) |
| **Post-intervention Quarters (ref: baseline all)** | | | | |
| 2016Q1 | 0.048 (0.044) | 0.014 (0.029) | -0.042 (0.044) | 0.011 (0.023) |
| 2016Q2 | 0.095* (0.052) | -0.013 (0.019) | -0.089** (0.040) | -0.010 (0.015) |
| 2016Q3 | 0.062 (0.049) | -0.010 (0.037) | -0.060 (0.040) | -0.009 (0.029) |
| 2016Q4 | 0.090** (0.046) | -0.038 (0.037) | -0.091** (0.040) | -0.030 (0.029) |
| 2017Q1 | 0.087 (0.062) | -0.045 (0.037) | -0.089* (0.049) | -0.034 (0.029) |
| 2017Q2 | -0.024 (0.058) | -0.067* (0.038) | 0.009 (0.060) | -0.053* (0.029) |
| 2017Q3 | 0.023 (0.088) | 0.018 (0.016) | -0.019 (0.083) | 0.015 (0.013) |
| 2017Q4 | 0.078 (0.066) | -0.058 (0.055) | -0.084 (0.053) | -0.046 (0.043) |
| **Interaction Effect (ref: Control at baseline all)** | | | | |
| PREDICT/2016Q1 | -0.034 (0.047) | -0.043 (0.030) | 0.025 (0.053) | -0.034 (0.024) |
| PREDICT/2016Q2 | -0.058 (0.050) | 0.020 (0.039) | 0.066 (0.052) | 0.016 (0.030) |
| PREDICT/2016Q3 | -0.065 (0.050) | -0.010 (0.044) | 0.067 (0.054) | -0.008 (0.034) |
| PREDICT/2016Q4 | -0.059 (0.052) | 0.007 (0.052) | 0.064 (0.060) | 0.006 (0.040) |
| PREDICT/2017Q1 | -0.136** (0.051) | 0.050 (0.036) | 0.169*** (0.067) | 0.039 (0.028) |
| PREDICT/2017Q2 | -0.022 (0.067) | 0.009 (0.043) | 0.024 (0.071) | 0.007 (0.034) |
| PREDICT/2017Q3 | -0.068 (0.068) | -0.054** (0.026) | 0.061 (0.074) | -0.042** (0.020) |
| PREDICT/2017Q4 | -0.045 (0.040) | -0.017 (0.058) | 0.043 (0.039) | -0.013 (0.045) |
| **Constant** | 72.314*** (9.463) | 2.015*** (0.255) | -0.987*** (0.002) | 67.407*** (4.499) |

Notes for TA3: Robust standard errors in parentheses; *** p<0.01, ** p<0.05, * p<0.1; Clustering on "county" to adjust for within-county homoskedasticity; Alpha test ZINB vs. ZIP = -0.919*** (0.007) [Significant means ZINB is preferred over ZIP]

### ***Table TA4. Use and Cost of Restorative Services: Difference-in-Differences Analyses***

| ***% Change Rate [exp(b)-1]*** | **Restorative: Diff-in-Diff (2016Q1 - 2017Q4 vs. Baseline All)** | | | |
| --- | --- | --- | --- | --- |
| Total # of Patient Quarters = 1,236,967 | **Utilization: ZINB model** | | **Cost: two-part model** | |
| Covariates | Inflate (0 vs. 1) | Count | Logit (1 vs. 0) | GLM |
| **Age (ref: 0 to 5)** |  |  |  |  |
| 6 to 12 | -0.486*** (0.009) | -0.304*** (0.012) | 0.508*** (0.024) | -0.219*** (0.009) |
| 13 to 18 | -0.504*** (0.009) | -0.375*** (0.013) | 0.470*** (0.019) | -0.265*** (0.010) |
| **Gender (ref: Female)** |  |  |  |  |
| Male | 0.007 (0.028) | -0.003 (0.016) | -0.008 (0.020) | -0.000 (0.010) |
| **Race/Ethnicity (ref: White)** |  |  |  |  |
| Hispanic Non-White | -0.073*** (0.019) | -0.085*** (0.015) | 0.020* (0.012) | -0.051*** (0.011) |
| Other | 0.167*** (0.039) | 0.035 (0.034) | -0.111*** (0.016) | 0.022 (0.022) |
| **Coverage Days (ref: <30 days)** |  |  |  |  |
| 30-59 days | -0.100* (0.054) | -0.112*** (0.038) | 0.037 (0.056) | -0.066** (0.031) |
| 60-89 days | -0.125*** (0.043) | -0.087 (0.052) | 0.077 (0.053) | -0.055 (0.041) |
| >= 90 days | -0.182*** (0.037) | -0.115** (0.042) | 0.124** (0.053) | -0.070** (0.032) |
| **Other county-level covariates** |  |  |  |  |
| Uninsured %Point (1-100) | -0.005 (0.008) | -0.004 (0.006) | 0.002 (0.005) | -0.003 (0.004) |
| # Dentist/100k population | -0.002 (0.001) | -0.002*** (0.001) | 0.000 (0.001) | -0.002*** (0.000) |
| Population/Square Miles | 0.002*** (0.001) | 0.002*** (0.000) | -0.001*** (0.000) | 0.001*** (0.000) |
| **PREDICT (ref: Control)** | -0.036 (0.038) | -0.057 (0.038) | 0.002 (0.029) | -0.034 (0.025) |
| **Post-intervention Quarters (ref: baseline all)** | |  |  |  |
| 2016Q1 | 0.050 (0.063) | -0.076 (0.079) | -0.076*** (0.019) | -0.045 (0.050) |
| 2016Q2 | 0.090*** (0.036) | -0.037 (0.066) | -0.089*** (0.017) | -0.021 (0.043) |
| 2016Q3 | 0.180*** (0.068) | 0.015 (0.058) | -0.127*** (0.032) | 0.019 (0.041) |
| 2016Q4 | 0.098** (0.052) | 0.065 (0.060) | -0.043* (0.026) | 0.036 (0.035) |
| 2017Q1 | 0.156* (0.091) | 0.112* (0.064) | -0.068 (0.062) | 0.035 (0.034) |
| 2017Q2 | 0.073 (0.105) | -0.020 (0.079) | -0.065 (0.063) | -0.045 (0.054) |
| 2017Q3 | 0.075 (0.106) | -0.082 (0.069) | -0.099 (0.064) | -0.063 (0.045) |
| 2017Q4 | 0.166* (0.097) | 0.040 (0.074) | -0.105* (0.056) | -0.006 (0.044) |
| **Interaction Effect (ref: Control at baseline all)** | |  |  |  |
| PREDICT/2016Q1 | -0.022 (0.067) | 0.064 (0.108) | 0.049* (0.029) | 0.037 (0.060) |
| PREDICT/2016Q2 | 0.054 (0.043) | 0.156* (0.088) | 0.031 (0.031) | 0.077 (0.053) |
| PREDICT/2016Q3 | 0.035 (0.071) | 0.103 (0.079) | 0.023 (0.043) | 0.049 (0.052) |
| PREDICT/2016Q4 | 0.044 (0.076) | -0.023 (0.075) | -0.049 (0.036) | -0.022 (0.046) |
| PREDICT/2017Q1 | -0.007 (0.071) | -0.022 (0.086) | -0.004 (0.054) | 0.006 (0.052) |
| PREDICT/2017Q2 | 0.088 (0.101) | 0.078 (0.081) | -0.037 (0.054) | 0.067 (0.058) |
| PREDICT/2017Q3 | 0.096 (0.080) | 0.222*** (0.087) | 0.024 (0.055) | 0.138*** (0.053) |
| PREDICT/2017Q4 | 0.033 (0.064) | 0.050 (0.076) | -0.003 (0.038) | 0.050 (0.047) |
| **Constant** | 9.742*** (1.913) | -0.042 (0.132) | -0.973*** (0.003) | 169.575*** (12.789) |

Notes for TA4: Robust standard errors in parentheses; *** p<0.01, ** p<0.05, * p<0.1; Clustering on "county" to adjust for within-county homoskedasticity; Alpha test ZINB vs. ZIP = 3.985*** (0.421) [Significant means ZINB is preferred over ZIP]
